# Functional Outcomes After Treatment of Posterior Inferior Cerebellar Artery Aneurysms

**DOI:** 10.7759/cureus.11746

**Published:** 2020-11-28

**Authors:** Mostafa Fatehi, Michael A Rizzuto, Swetha Prakash, Charles Haw, Peter A Gooderham, Gary J Redekop

**Affiliations:** 1 Division of Neurosurgery, Vancouver General Hospital, Vancouver, CAN

**Keywords:** endovascular aneurysm repair, microsurgical aneurysm clipping, posterior inferior cerebellar artery, subarachnoid hemorrhage

## Abstract

Objective

Aneurysms of the posterior inferior cerebellar artery (PICA) are a rare cause of subarachnoid hemorrhage. Treatment for this type of aneurysm may be microsurgical clipping or endovascular. This decision is based on patient characteristics, aneurysm location and dimensions, along with surgeon and institutional experience. In this study we aim to assess the outcomes of surgical and endovascular treatment of PICA aneurysms.

Methods

We retrospectively reviewed the charts of 52 patients who were admitted to Vancouver General Hospital for ruptured or symptomatic PICA aneurysms between 2005 and 2015. Modified Rankin scores were assigned at the time of discharge and at two subsequent follow-up time points. The mean short-term follow-up period post-operatively was 11.1 months and the mean long-term follow-up period was 19.3 months. Clinical and radiological characteristics were collected for all patients.

Results

Of the 52 patients, two died prior to obtaining treatment. Of the 50 patients who were treated for their PICA aneurysm, 39 presented with subarachnoid hemorrhage while 11 had symptomatic unruptured PICA aneurysms. Overall, 11 patients had endovascular treatment (coil embolization) while 39 patients underwent microsurgical clipping/trapping of the aneurysm. At the time of hospital discharge, patients in the microsurgical group trended towards a better the modified Rankin Scale score (2.3) compared to the endovascular group, though this did not reach significance (3.0) (p=0.20). The long-term score in the endovascular group (1.6) was also comparable to the microsurgical group (1.9) (p=0.55).

Conclusion

While the early outcomes in patients treated endovascularly appear better, there is no statistically significant difference in outcomes between the microsurgical and endovascular treatment groups at short- and long-term follow-up.

## Introduction

Aneurysms of the posterior inferior cerebellar artery (PICA) are a rare cause of subarachnoid hemorrhage and account for 0.5-3% of all intracranial aneurysms [1-2]. Treatment for ruptured and symptomatic PICA aneurysms may be microsurgical clipping [3-5] or endovascular (including coil embolization, flow diverting stent reconstruction, and occasionally proximal occlusion) [6-8]. Treatment choice is affected by patient characteristics, aneurysm location and dimensions, as well as surgical and institutional experience. Furthermore, treatment is challenged by a high proportion of fusiform aneurysms, the tortuosity of the PICA, and relative proximity to lower cranial nerves in the posterior fossa [9]. Consequently, it was previously reported that patients with ruptured PICA aneurysms had a relatively poor clinical outcome [10-12].

Due to their rarity, there is a dearth of high-quality outcome studies for PICA aneurysms. Two large studies that attempted to assess outcomes for ruptured PICA aneurysms are the Barrow Ruptured Aneurysm Trial (BRAT) [12] and International Subarachnoid Aneurysm Trial (ISAT) [13]. These represent two of the largest aneurysm trials published. Nevertheless, conclusions from these studies guiding the treatment of PICA aneurysms are limited by the small sample size of the PICA cohort. To date, several case series have reported variable treatment outcomes with either treatment modality. However, there is a scarcity of data regarding long-term, functional outcomes for PICA aneurysms.

More recently, two groups have published retrospective analyses of their patients with PICA aneurysms. The first, by Bohnstedt et al. [10], reported the early post-treatment outcomes for PICA or vertebral artery aneurysms and found that the most significant predictors of outcome were age and presenting Hunt-Hess (HH) score and not aneurysm location or management modality. However, they also noted significantly higher complications after clipping compared to endovascular treatment. A multicenter registry review from Switzerland reported functional outcomes for 62 patients [11]. Two-thirds of these patients underwent endovascular treatment and the authors found generally good outcomes at discharge or one-year follow-up. However, the effect of treatment choice was not reported in this study.

In this study, we present the clinical characteristics and functional outcomes of patients undergoing clipping or coiling for ruptured and unruptured PICA aneurysms at Vancouver General Hospital (VGH) from 2005 to 2015 with the aim of determining if outcomes between treatment modalities are equivalent.

## Materials and methods

This study is a single-center retrospective review of a prospectively maintained database of patients admitted to VGH with unruptured or ruptured PICA aneurysms. Due to advances in endovascular management in the past two decades, data collection was limited to patients admitted between 2005 and 2015. From the VGH Neurosurgical Database, 52 patients with PICA aneurysms were identified. Variables collected include patient demographics, clinical presentation, Hunt-Hess scores, treatment complications, radiological characteristics, and hospital length of stay (LOS). Long-term outcomes were obtained by reviewing the charts of treating neurosurgeons. A score on the modified Rankin scale (mRS) was assigned for each patient at the time of hospital discharge and at follow-up appointments with their treating surgeon. Post-operative follow-up visit timelines vary in each surgeon’s practice. The “short-term follow-up” timepoint in this study was the first post-operative appointment where mRS was recorded while the “long-term follow-up” timepoint was approximately one year later. A simple non-parametric T-test was used to compare mean mRS scores and clinical characteristics over time, between treatment modalities, and between ruptured and unruptured presentations (Prism 7, Graphpad Software, La Jolla, CA, USA, ). This study was approved by the University of British Columbia Clinical Research Ethics Board, approval #H17-02147.

A total of 52 patients were admitted with unruptured or ruptured PICA aneurysms during the study period. Of the 52 patients, two patients with subarachnoid hemorrhage (SAH) died prior to the treatment of their PICA aneurysm. There was a strong predilection toward females presenting with ruptured aneurysms (Table 1). The mean age at presentation was 59.3 (SD 13.8) years old. Of the 50 treated patients, 39 presented with SAH and 11 were treated for symptomatic unruptured PICA aneurysms. Eleven patients had endovascular treatment while 39 patients underwent microsurgical clipping/trapping of the aneurysm.

**Table 1:** Patient demographics and presentation characteristics PICA: posterior inferior cerebellar artery, SAH: subarachnoid hemorrhage

	Total	Ruptured	Unruptured	p
Number Treated	50	39	11	
Sex				
Male	16	15	1	
Female	34	24	10	<0.05
Age				
Mean (SD)	59.3 (13.8)	58.8 (14.1)	61.3 (13.5)	0.6
Location on PICA				
Distal	30	24	6	
Proximal	20	15	5	
Hunt-Hess SAH Grade				
I	0	0	-	
II	9	9	-	
III	13	13	-	
IV	11	11	-	
V	2	2	-	
Not listed	4	4	-	

## Results

The majority of all treated aneurysm were occluded based on post-operative CT angiogram (Table 2). The mean hospital length of stay (LOS) was equivalent in the endovascular and microsurgical groups. When stratified by presentation, patients with ruptured aneurysms required more time in hospital (Table 3, Table 4). There was no difference between treatment modalities or treatment modality within all ruptured presentations when comparing the number of patients that were discharged home and those that were transferred to their local hospital or rehabilitation facility. However, a greater proportion of patients with unruptured aneurysms were discharged home directly after treatment as compared to those presenting with SAH. Among all 50 patients, retreatment was only required for an unruptured aneurysm treated endovascularly. Functional outcomes characterized by modified Rankin scale scores at discharge were no different in the microsurgical group (2.3) compared to the endovascular group (3.0). This remained the case at one-year follow-up (mean=11.1 months) in the endovascular group (1.4) and microsurgical group (2.0). At long-term follow-up (mean=19.3 months), mRS remained equivalent between the endovascular (1.6) and microsurgical groups (1.9) (Table 2). When stratifying patients by ruptured or unruptured presentation (Table 3) or treatment modality within the ruptured group (Table 4), mRS scores at all time points remained equivalent.

**Table 2:** Outcomes stratified by treatment modality

	Microsurgical	Endovascular	p
Number Treated	39	11	
Degree of Occlusion			
Complete	35	9	
Residual Neck	2	1	
Residual Aneurysm	2	1	
Retreatment	0	1	0.34
Hospital Length of Stay			
Mean days (SD)	28.3 (40.5)	22.7 (19.8)	0.53
Modified Rankin Score			
Mean at discharge (SD)	2.3 (1.2)	3.0 (1.3)	0.20
	n=32	n=7	
Mean at short-term follow-up (SD)	2.0 (1.2)	1.4 (0.8)	0.12
	n=31	n=7	
Mean at long-term follow-up (SD)	1.9 (1.0)	1.6 (0.9)	0.55
	n=19	n=5	
Disposition			
Home	23	5	0.54
Rehabilitation facility	4	1	
Local hospital	5	2	
Unknown	6	2	
Mortality	1	1	

**Table 3:** Outcomes stratified by presentation

	Ruptured	Unruptured	p
Number Treated	39	11	
Degree of Occlusion			
Complete	36	8	
Residual Neck	1	2	
Residual Aneurysm	2	1	
Retreatment	0	1	0.34
Hospital Length of Stay			
Mean days (SD)	31.6 (40.5)	11.3 (8.0)	<0.05
Modified Rankin Score			
Mean at discharge (SD)	2.5 (1.3)	2.1 (1.2)	0.40
	n=29	n=10	
Mean at short-term follow-up (SD)	1.9 (1.2)	1.9 (1.0)	0.89
	n=29	n=8	
Mean at long-term follow-up (SD)	1.9 (1.0)	1.4 (1.1)	0.37
	n=19	n=5	
Disposition			
Home	18	10	<0.05
Rehabilitation facility	6	0	
Local hospital	5	1	
Unknown	8	0	
Mortality	2	0	

**Table 4:** Outcomes in ruptured presentations stratified by treatment modality

	Ruptured Microsurgical	Ruptured Endovascular	p
Number Treated	31	8	
Degree of Occlusion			
Complete	29	7	
Residual Neck	1	0	
Residual Aneurysm	1	1	
Retreatment	0	0	
Hospital Length of Stay			
Mean days (SD)	32.6 (44.4)	27.5 (20.7)	0.64
Modified Rankin Score			
Mean at discharge (SD)	2.4 (1.2)	3.0 (1.4)	0.40
	n=24	n=5	
Mean at short-term follow-up (SD)	2.0 (1.2)	1.6 (0.9)	0.42
	n=24	n=5	
Mean at long-term follow-up (SD)	2.0 (1.0)	1.74 (1.0)	0.67
	n=15	n=4	
Disposition			
Home	16	2	0.43
Rehabilitation facility	4	1	
Local hospital	4	2	
Unknown	6	2	
Mortality	1	1	

Major complications included stroke, dysphagia, development of hydrocephalus, seizure and meningitis; complications classified as minor were urinary tract infections, hyponatremia, delirium and voice hoarseness (Table 5). Major complications were of similar incidence in ruptured presentations compared to unruptured presentations. Additionally, treatment modality did not impact development of major complications or minor complications. Minor complications were more common in patients with ruptured presentations as compared to unruptured presentations.

**Table 5:** Complications by treatment modality and rupture status

Complication	Microsurgical (n=39)		Endovascular (n=11)		p
	Ruptured	Unruptured	Ruptured	Unruptured	
Major					
Death	1	0	1	0	
Stroke	2	1	1	0	
Cerebral	1	1	0	0	
Cerebellar	1	0	1	0	
Dysphagia	2	0	0	1	
Hydrocephalus	3	0	2	0	
Seizure	1	0	0	0	
Meningitis	1	0	0	0	
Microsurgical Subtotal	11		Endovascular Subtotal	5	0.45
Ruptured Subtotal	14		Unruptured Subtotal	2	0.25
Minor					
Urinary Tract Infection	3	0	0	0	
Voice Hoarseness	2	0	0	0	
Pneumonia	3	0	2	0	
Hyponatremia	3	0	1	0	
Delirium	0	0	2	0	
Microsurgical Subtotal	11		Endovascular Subtotal	5	0.34
Ruptured Subtotal	16		Unruptured Subtotal	0	<0.05

## Discussion

Aneurysms of the posterior inferior cerebellar artery are a relatively uncommon cause of subarachnoid hemorrhage. Their treatment is complicated by a multitude of factors including but not limited to, close proximity to cranial nerves and the brainstem as well as higher rates of hemorrhage. The literature suggests that PICA aneurysms have worse functional outcomes as compared to other cerebral aneurysms [4,12-14]. However, there is limited existing literature regarding differences in outcomes when comparing treatment modality for PICA aneurysms.

With 31 ruptured PICA aneurysms in the study, the multicenter ISAT trial did not include a sufficient number of PICA aneurysms to yield a meaningful conclusion on their management. Patients were assessed at two months and one year post-operatively, but it is unclear how many of the 31 PICA patients were included at each timepoint [6].

The BRAT trial included 21 ruptured PICA aneurysms and found that despite presenting with similar Hunt-Hess (HH) [15] scores to other posterior circulation aneurysms, PICA aneurysms were significantly associated with poorer outcomes (modified Rankin scale score >2). When compared to all non-PICA aneurysms at the time of discharge, one-year, three-year, and six-year follow-up, patients with PICA aneurysms consistently presented with statistically significant worse functional outcomes. At the six-year follow-up, 63% of PICA aneurysm patients had a poor outcome compared to 37% of non-PICA aneurysm patients [12]. The authors reported that this disparity may be attributed to the disproportionate proportion of PICA aneurysms randomized to the microsurgical arm (86%). The BRAT trial concluded that much like treatment for other aneurysms, the choice of surgical vs. endovascular treatment for PICA aneurysms depends on patient characteristics and clinician expertise [14]. At one-year follow-up the BRAT demonstrated superiority of endovascular treatment for ruptured posterior circulation aneurysms with respect to functional outcomes [12]. However, in keeping with our findings the BRAT demonstrated no difference between treatment modalities at three-, six-, and 10-year follow-up [14].

The present study is one of the largest single-institution reviews of treatment for PICA aneurysms to date. We present outcomes in a large series of patients that have undergone treatment for PICA aneurysms. Our institutional experience is fairly unique among North American centers in the preponderance of surgical management. Analysis of the present data suggests that while there are better short-term outcomes in patients treated endovascularly, there is no difference in mRS score between endovascular and microsurgical treatment of ruptured PICA aneurysms at follow-up. A favourable mRS has typically been defined as a score of 0 to 2, and we see that in our cohort of patients surviving to follow-up there is a favourable outcome on average [10-12,16]. There was in fact a single patient with mRS of 4 at long-term follow-up in the microsurgically treated group. In comparing our findings to the literature, we find that microsurgical clipping techniques are in fact a safe and viable treatment choice for PICA aneurysms as demonstrated by a few retrospective series [10,11,16].

When comparing ruptured PICA aneurysms to unruptured aneurysms we expectedly find that patients with SAH tend to have a longer hospital stay as they require extensive medical attention, long-term observation, and fewer patients are able to transition home at the time of discharge due to rehabilitation needs. Patients with SAH see a greater rate of minor complications compared to patients with unruptured aneurysms, which can likely be attributed to the secondary effects of SAH and the prolonged hospital stay required for these patients.

Due to the retrospective nature of the current study, a robust data set was not available with some data having not been collected and thus certain features could not be subjected to analysis. Additionally, analysis of our data is limited by the small population size. This potentially affects our ability to detect statistical differences between treatment groups. Given the sample size, the differences reported here may be influenced by patient comorbidities which potentially influenced treatment choice and may have predisposed certain patients to post-operative complications.

While PICA aneurysms are technically challenging to treat microsurgically given their proximity to lower cranial nerves and proximal segment of the PICA, it is often the preferred approach in aneurysms with challenging architecture. One might opt for a microsurgical approach in those aneurysms with a wide neck, fusiform appearance or those with a dissected or hypoplastic proximal segment or vertebral artery limiting endovascular access. Individual patient and aneurysm characteristics as well as surgeon experience may well have the greatest influence on outcomes when treating patients with PICA aneurysms.

## Conclusions

Overall, functional outcomes after treatment of PICA aneurysms by endovascular or microsurgical means at Vancouver General Hospital over the 10-year period in question were equivalent at all follow-up time points. Patient disposition from hospital and average length of stay were also equivalent between the treatment modalities. As expected, patients presenting with ruptured PICA aneurysms were in hospital for significantly longer and discharged directly home to a lesser extent than those presenting in an unruptured fashion. Our study supports the notion that the treatment of PICA aneurysms should be decided based on patient characteristics, aneurysm architecture and surgeon experience as treatment modalities appear equivalent.
